# RETRACTED: Refolo et al. Chlorogenic Acid Improves the Regorafenib Effects in Human Hepatocellular Carcinoma Cells. *Int. J. Mol. Sci.* 2018, *19*, 1518

**DOI:** 10.3390/ijms27052197

**Published:** 2026-02-26

**Authors:** Maria Grazia Refolo, Catia Lippolis, Nicola Carella, Aldo Cavallini, Caterina Messa, Rosalba D’Alessandro

**Affiliations:** Laboratory of Cellular and Molecular Biology, Department of Clinical Pathology, National Institute for Digestive Diseases, IRCCS “Saverio de Bellis”, Via Turi 27, 70013 Castellana Grotte, BA, Italy; maria.refolo@irccsdebellis.it (M.G.R.); catiuscia85@hotmail.it (C.L.); nicola.carella@irccsdebellis.it (N.C.); cavaldo@libero.it (A.C.)

The Journal retracts the article “Chlorogenic Acid Improves the Regorafenib Effects in Human Hepatocellular Carcinoma Cells” [1] cited above.

Following publication, concerns were brought to the attention of the Editorial Office regarding the integrity of several images presented in this publication [1].

In accordance with the journal’s standard procedures, the Editorial Office and Editorial Board conducted an investigation that confirmed a duplication between sub-panels in Figure 2, as well as a partial duplication of Western blots within Figure 5 and between Figure 5 and Figure 7 in [2], produced by a similar author group, presenting different experimental conditions. The authors fully cooperated with the investigation and indicated that an error had occurred in the collation of the figures. However, as the original images could not be provided for verification, the Editorial Board and the authors were unable to resolve this issue and confirm the reliability of this study. As a Consequence, the Editorial Board has lost confidence in the reliability of the findings and decided to retract this publication [1], as per MDPI’s retraction policy.

This retraction was approved by the Editor-in-Chief of the journal *IJMS*.

The authors have been informed of this retraction but did not provide a comment on this decision.
